# Indifference of Patients

**Published:** 1897-10

**Authors:** 


### INDIFFERENCE OF PATIENTS.

In the daily drudge of the over-worked doctor, there is seen so much indifference on the part of patients and their relatives and friends that it is not to be wondered at, if he gets a shade careless and indifferent. The wonder would be if he did not. When he finds in his work day by day that his advice is disregarded, that his instructions are not carried out, that his medicines are not administered and that his patient as well as relatives are indifferent to the final result, it is enough to drive a sensitive man entirely out of the practice of medicine.

It has always been a surprise to us that as many men practice medicine as do. Probably if a man could change his profession like he changes his coat, we would find but few men to care for the sick and ailing.

There has recently occured to us three examples of such dreadful indifference on the part of patients and their friends that a few more would be sufficient to drive us to seek fame and fortune in other fields. A woman consulted us early this month for a pulmonary hemorrhage. She had been under our care for pulmonary tuberculosis and had seen one of her friends die of a hemorrhage. She was of moderate intelligence and had fairly well followed our instructions up to this time. She came in for an office consultation bringing with her a pint cup which was three-fourths full of blood, which she had coughed up on the street. She was told to return home on the cable car at once, go to bed immediately and to take some medicine which was telephoned for. She was told she might spit up more blood and that she must be very careful as her chest was full of bubbling rales. This was at four o'clock; at seven the same woman was seen carrying a large basket of vegetables, running ten feet to catch the car which she should have taken three hours before. That night she had another very severe hemorrhage and on being called, we refused to go. She got another physician and after a week or two in bed, made a recovery.

Following this we were called to see a child with catarrhal pneumonia in a family of young children. The room was ordered to be kept warm, a jacket to be applied and stimulants and some other combination to be administered. As the child was quite ill and had been so for several days, the mother was told the seriousness of the case and that the child would have to receive the best of care if it recovered. For twenty-four hours the treatment was carried on faithfully and well. The next visit was made on the second day at an unusual hour, and lo and behold the mother had gone up town to attend a "fire sale" leaving a twelve-year-old daughter to attend to the baby. She had been away for two and a half hours at the time of our visit. We stormed and we swore and at present that baby is getting the attention that it demands.

The third case is equally as bad as the others. A little child with both ears running profusely came under our care, and syringing with subsequent insufflation of boracic acid was ordered every three hours. The baby was brought back several times and "no improvement" was the report each time. Being in the immediate neighborhood of their residence several days later a visit was made on the child at 11:45 A. M. Both ears were found to be full of pus and the mother confessed that the child's ears had received no attention since 4 P. M. of the previous day.

Again we say that a physician has a right to get careless and indifferent. If people don't care, why should the doctor? Only personal ambition and the desire to give return for money to be received is in such cases the mainspring of action. It is lucky for such indifference if the attending physician has ambition. Many patients are not at all pleasing and with a tired doctor and an indifferent patient, Providence must rule. —*Colorado Medical Journal.*

